# Post Hoc, Sex‐Specific Subgroup Analysis of Efgartigimod in Patients With Generalized Myasthenia Gravis From the ADAPT Trial: A Sex and Gender Equity in Research (SAGER) Guidelines Approach

**DOI:** 10.1002/mus.70135

**Published:** 2026-01-10

**Authors:** Sarah Hoffmann, Sihui Zhao, Filip Callewaert, Silke Schoppe, Csilla Rózsa, Jennifer Spillane

**Affiliations:** ^1^ Department of Neurology Neuroscience Clinical Research Center (NCRC) and Integrated Myasthenia Gravis Center, Charité‐Universitätsmedizin Berlin Germany; ^2^ Argenx Ghent Belgium; ^3^ Department of Neurology, Jahn Ferenc Dél‐Pesti Hospital Budapest Hungary; ^4^ Centre for Neuromuscular Disease, National Hospital for Neurology and Neurosurgery Queen Square University College London Hospitals (UCLH) National Health Service (NHS) Foundation Trust London UK

**Keywords:** autoantibodies, efgartigimod alfa, gender, generalized myasthenia gravis, Phase 3 clinical trial, sex differences, therapeutic response

## Abstract

**Introduction/Aims:**

Sex‐specific differences in myasthenia gravis (MG) are widely acknowledged, yet data on sex‐based outcomes of MG treatment are scarce. In accordance with Sex and Gender Equity in Research guidelines, this post hoc analysis assessed potential sex‐specific differences in treatment outcomes in acetylcholine receptor antibody–positive (AChR‐Ab+) generalized (g)MG participants in the Phase 3 ADAPT trial (NCT03669588).

**Methods:**

Participants received four once‐weekly efgartigimod infusions (10 mg/kg) or placebo per cycle. Endpoints (primary: proportion of Myasthenia Gravis Activities of Daily Living (MG‐ADL) responders (Cycle 1); secondary: proportion of Quantitative Myasthenia Gravis (QMG) and early (Cycle 1) MG‐ADL responders, and time with clinically meaningful improvements in MG‐ADL score; additional: quality of life outcomes, pharmacodynamics) were assessed according to sex.

**Results:**

Females were younger (mean age: 42.9 vs. 54.8 years), more likely to have undergone thymectomy (65.1% [56/86] vs. 44.2% [19/43]), and had higher baseline QMG scores (16.3 vs. 14.3) compared with males. Efgartigimod demonstrated homogeneous effects between sexes, with no significant difference in proportions of MG‐ADL (*p* = 0.7014), early (Cycle 1) MG‐ADL (*p* = 1.00), or QMG responders (*p* = 0.1595). Improvements in quality‐of‐life assessments, rates of minimal symptom expression, and mean total immunoglobulin G reductions (Cycle 1) were greater with efgartigimod verso placebo in females and males. Efgartigimod was well tolerated, with similar safety profiles across sexes.

**Discussion:**

In ADAPT, efgartigimod‐treated female and male AChR‐Ab+ gMG patients had similar efficacy and safety outcomes. These data provide valuable insight for clinicians, given the established sex differences in MG disease course and treatment responses.

**Trial Registration:** The ADAPT trial is registered on ClinicalTrials.gov (NCT03669588).

**Abbreviations**
AChE: acetylcholinesterase
AChR‐Ab+: acetylcholine receptor antibody–positive
ANCOVA: analysis of covariance
BMI: body mass index
CI: confidence interval
CMI: clinically meaningful improvement
EFG: efgartigimod
gMG: generalized myasthenia gravis
IgG: immunoglobulin G
IV: intravenous
LS: least‐squares
LSMD: least‐squares mean difference
MG: myasthenia gravis
MG‐ADL: Myasthenia Gravis Activities of Daily Living
MGC: Myasthenia Gravis Composite
MGFA: Myasthenia Gravis Foundation of America
MG‐QoL15r: 15‐item revised Myasthenia Gravis Quality of Life
MSE: minimal symptom expression
NSIST: nonsteroidal immunosuppressive therapy
OR: odds ratio
PBO: placebo
PI: principal investigator
QMG: Quantitative Myasthenia Gravis
QoL: quality of life
SAGER: Sex and Gender Equity in Research
SE: standard error
TEAE: treatment‐emergent adverse event

## 1 Introduction

Epidemiologic and clinical indicators point to a relevant role of sex in myasthenia gravis (MG), with significant differences observed between females and males in disease course, severity, and treatment response across multiple observational and retrospective studies [1, 2, 3, 4, 5, 6, 7]. Females more frequently experience early‐onset MG, a higher rate of thymic hyperplasia, a higher risk of myasthenic exacerbations, and a higher disease severity, and they tend to have a lower quality of life (QoL) compared with males [1, 2, 3, 5, 6]. Previous studies have demonstrated sex‐specific differences in response to conventional MG treatments, including a more recent Phase 3, randomized, placebo‐controlled study with nipocalimab [8], with females improving less than males in both objective and patient‐reported outcomes [4, 7, 9]. These sex differences in outcomes have been observed in both refractory and nonrefractory patients and occurred despite comparable treatment dosing between males and females [7, 8].

Whilst the Sex and Gender Equity in Research (SAGER) guidelines recommend a systematic approach to the reporting of sex and gender in clinical trials [10], these are often overlooked in the design, implementation, and reporting of clinical trials, thus limiting the generalizability of research findings and their applicability to clinical practice [10]. This is of particular importance in generalized MG (gMG), a condition with evidence of sex‐specific differences in the underlying mechanisms of the disease [1, 4, 7]. Yet, studies completed to date assessing sex differences in gMG have been observational or retrospective in study design, limiting the data quality and the conclusions that can be drawn [1, 2, 3, 4, 6, 7].

Efgartigimod is a human immunoglobulin (Ig) G1 antibody Fc fragment that blocks the neonatal Fc receptor, thereby decreasing IgG levels, including those of pathogenic IgG autoantibodies, which play a key role in the pathogenesis of gMG [11, 12, 13]. The pivotal, Phase 3 ADAPT trial (NCT03669588) evaluated the efficacy and safety of intravenous (IV) efgartigimod treatment in adult participants with gMG. The primary data demonstrated that efgartigimod was well tolerated and resulted in clinically meaningful improvements (CMIs) in gMG‐specific outcome measures in participants with acetylcholine receptor antibody–positive (AChR‐Ab+) gMG compared with participants who received placebo [12]. These data led to the approval of efgartigimod in the United States and Europe for the treatment of adults with gMG who are AChR‐Ab+ [14, 15] and in Japan for the treatment of adults with gMG who do not have sufficient response to steroids or nonsteroidal immunosuppressive therapies, regardless of antibody status [16]. This post hoc subgroup analysis aimed to identify any potential sex‐specific differences in outcomes, safety profiles, and pharmacodynamics between female and male participants who received efgartigimod or placebo within the ADAPT trial.

## 2 Methods

### 2.1 Study Design and Patients

The design of the ADAPT trial (NCT03669588) has been described previously [12]. Briefly, ADAPT was a randomized, double‐blind, placebo‐controlled, multicenter, Phase 3 trial in participants with gMG. Participants were randomized 1:1 to IV efgartigimod (10 mg/kg) or matching placebo treatment groups and received an initial treatment cycle of four once‐weekly infusions. Subsequent treatment cycles were administered according to individual clinical response. Efficacy assessments included Myasthenia Gravis Activities of Daily Living (MG‐ADL scale) [17], Quantitative Myasthenia Gravis (QMG) [18], and 15‐item revised Myasthenia Gravis Quality of Life (MG‐QoL15r) [19].

### 2.2 Ethics Statement

The trial was conducted in accordance with the principles outlined in the Declaration of Helsinki. Independent ethics committees and international review boards provided written approval for the study protocol and all amendments. We confirm that we have read the journal's position on issues involved in ethical publication and affirm that this report is consistent with those guidelines.

### 2.3 Data Definitions and Outcomes

For this post hoc subanalysis of the ADAPT trial according to sex, only outcomes from AChR‐Ab+ participants were assessed, due to the low number of ADAPT study participants positive for muscle‐specific kinase antibodies (6 of 167 enrolled participants) [12]. Sex was defined as “sex at birth,” as self‐reported by the patient, with the available binary options of male or female. The primary endpoint, selected secondary endpoints (that demonstrated significant [adjusted *p* < 0.05] efgartigimod treatment effect compared with placebo in the hierarchical testing sequence in the primary ADAPT analysis), and selected exploratory endpoints from the ADAPT trial were used in this post hoc subanalysis. In the ADAPT trial, the primary endpoint was the proportion of AChR‐Ab+ participants who were MG‐ADL responders in the first treatment cycle, defined as a ≥ 2‐point improvement (reduction) in MG‐ADL score, sustained for ≥ 4 consecutive weeks, with the first improvement occurring no later than 1 week after the last infusion. Secondary endpoints included (1) proportion of QMG responders (defined as a ≥ 3‐point improvement in the total QMG score for ≥ 4 consecutive weeks, with the first improvement occurring no later than 1 week after the last infusion) after the first treatment cycle; (2) percentage of time participants showed a (CMI) in MG‐ADL score (defined as a ≥ 2‐point reduction in total MG‐ADL score), up to and including Day 126; and (3) proportion of early MG‐ADL responders in Cycle 1 (defined as MG‐ADL responders with first MG‐ADL improvement of ≥ 2 points occurring by Week 2). Primary and secondary endpoints were tested in hierarchical order [12]; testing was stopped at “early MG‐ADL responder” due to the preceding endpoint not reaching significance (median time from Day 28 until no CMI; *p* = 0.26). Exploratory endpoints included change in MG‐ADL, QMG, and MG‐QoL15r scores and minimum point improvements in MG‐ADL and QMG scores during Cycle 1. Minimal symptom expression (MSE; defined as achieving an MG‐ADL total score of 0 or 1 at any post‐baseline visit) was assessed during Cycle 1 in all participants according to sex. Validated assays of total IgG were used to analyze pharmacodynamic effects. Safety was assessed through incidence of treatment‐emergent adverse events (TEAEs).

### 2.4 Statistical Analyses

This post hoc subanalysis was primarily based on descriptive statistical methods. Differences in baseline demographics, disease characteristics, and MG therapies between sex subgroups were tested using chi‐square or Fisher's exact test for categorical variables or two‐sample *t*‐test for continuous variables. Efficacy analyses were conducted in the modified intention‐to‐treat population and included participants who had a valid baseline and ≥ 1 post‐baseline MG‐ADL assessment, stratified by sex. For the categorical response variables, Zelen's exact test for homogenous/equal odds ratios for each sex subgroup was used owing to the small sample size, where the null hypothesis is that the odds ratio between efgartigimod and placebo in the female participants is the same as the odds ratio between efgartigimod and placebo in the male participants. For the continuous response variables, the coefficient of interaction term of treatment by sex was tested using the analysis of covariance (ANCOVA) model, with treatment, sex, treatment by sex, baseline values, and two stratification factors (Japanese vs. non‐Japanese, non‐steroidal anti‐inflammatory drug [NSID] user vs. non‐NSID user at baseline) as the explanatory variables. The endpoints were tested at the two‐sided 5% alpha level without multiple testing corrections. An exploratory sensitivity analysis using logistic regression or the ANCOVA model adjusted by age was used to assess the impact of age as a potential confounder on the homogeneity of treatment effect. Mixed model for repeated measurements (MMRM) analysis, with treatment, visit, sex, treatment by visit, treatment by sex, visit by sex, and treatment by visit by sex interaction as fixed effects, and baseline QMG score and stratification factors at randomization (Japanese/non‐Japanese and NSID use) as covariates, was used to model change from baseline of QMG scores at weeks 1 to 8. Within‐subject correlation was modeled by assuming an unstructured covariance matrix for the error terms. Safety analyses were conducted in participants who received ≥ 1 dose or part‐dose of study medication, stratified by sex.

## 3 Results

### 3.1 Patient Characteristics

Overall, 129 participants with AChR‐Ab+ gMG were included in this subanalysis. Participant demographics and baseline disease characteristics by sex at birth are shown in Table 1. No significant differences in disease severity were observed between female and male participants as measured by MG‐ADL, MG‐QoL, and MGFA classification. Female participants had a significantly higher mean QMG score at baseline compared with male participants.

**TABLE 1:** Demographics and baseline disease characteristics in the overall population and by sex at birth.

	Overall (*N* = 129)	Female (*n* = 86)	Male (*n* = 43)	Between‐sex subgroup analysis
Age, mean (SD), years	46.9 (15.4)	42.9 (14.3)	54.8 (14.5)	*p* < 0.0001
Time since diagnosis, mean (SD), years	9.30 (8.21)	9.95 (8.40)	8.02 (7.74)	*p* = 0.1981
Weight, mean (SD), kg	80.57 (25.17)	70.19 (15.67)	101.33 (27.78)	*p* < 0.0001
BMI, mean (SD), kg/m^2^	28.11 (7.1)	26.28 (6.13)	31.77 (7.57)	*p* < 0.0001
Previous thymectomy for gMG, *n* (%)	75 (58.1)	56 (65.1)	19 (44.2)	*p* = 0.0366
MGFA class at screening, *n* (%)				*p* = 0.4496
II	53 (41.1)	35 (40.7)	18 (41.9)	
III	71 (55.0)	49 (57.0)	22 (51.2)	
IV	5 (3.9)	2 (2.3)	3 (7.0)	
Total MG‐ADL score at baseline				
Mean (SD)	8.8 (2.3)	8.8 (2.2)	8.8 (2.6)	*p* = 0.9200
Median (range)	9.0 (5–16)	9.0 (5–15)	9.0 (5–16)	
Total QMG score at baseline, median (range)				
Mean (SD)	15.6 (4.8)	16.3 (4.8)	14.3 (4.4)	*p* = 0.0213
Median (range)	16 (4–28)	17.0 (4–28)	14.0 (6–26)	
Total MGC score at baseline, median (range)				
Mean (SD)	18.4 (5.6)	18.9 (5.6)	17.3 (5.6)	*p* = 0.1197
Median (range)	19.0 (3–33)	19.0 (3–33)	16.0 (8–31)	
Total MG‐QoL15r score at baseline				
Mean (SD)	16.2 (5.9)	16.0 (5.7)	16.4 (6.3)	*p* = 0.7287
Median (range)	17.0 (3–29)	16.5 (4–27)	18.0 (3–29)	
NSIST use during screening period, *n* (%)	77 (59.7)	53 (61.6)	24 (55.8)	*p* = 0.3428
Steroid use during screening period, *n* (%)	97 (75.2)	66 (76.7)	31 (72.1)	*p* = 0.7891
AChE inhibitor use during screening period, *n* (%)	114 (88.4)	73 (84.9)	41 (95.3)	*p* = 0.2665

Abbreviations: AChE, acetylcholinesterase; BMI, body mass index; gMG, generalized myasthenia gravis; MG‐ADL, Myasthenia Gravis Activities of Daily Living; MG‐QoL15r, 15‐item revised Myasthenia Gravis Quality of Life; MGC, Myasthenia Gravis Composite; MGFA, Myasthenia Gravis Foundation of America; NSIST, nonsteroidal immunosuppressive therapy (azathioprine, cyclosporine, cyclophosphamide, methotrexate, mycophenolate mofetil, mycophenolate sodium, tacrolimus); QMG, Quantitative Myasthenia Gravis; SD, standard deviation.

### 3.2 Efficacy

**FIGURE 1:**
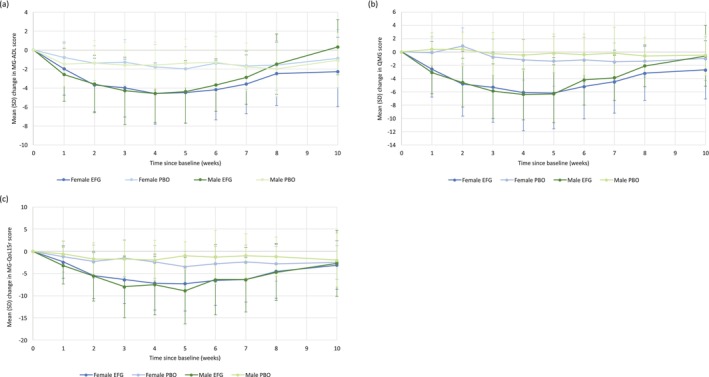
Mean change in (a) total MG‐ADL score, (b) QMG score, and (c) MG‐QoL15r score during Cycle 1 in the modified intent‐to‐treat population, by sex at birth. Error bars represent SD. EFG, efgartigimod; MG‐ADL, Myasthenia Gravis Activities of Daily Living; MG‐QoL15r, Myasthenia Gravis Quality of Life Questionnaire; PBO, placebo; QMG, Quantitative Myasthenia Gravis; SD, standard deviation.

No significant sex‐specific treatment differences were observed in the primary endpoint (proportion of MG‐ADL responders) or key secondary endpoints (percentage of time participants showed CMI in MG‐ADL score, up to Day 126; or proportion of early MG‐ADL responders in Cycle 1) (Table 2) or Cycle 2 (Table S1). Sex‐specific treatment effects on the proportion of QMG responders were numerically more pronounced but not statistically significant (Table 2). However, MMRM for change from baseline found no sex‐specific treatment effects over the first 8 weeks of Cycle 1 (Tables S2 and S3). The sensitivity analysis using models adjusted by age did not show a significant interaction of treatment by sex (data not shown). Comparable magnitudes of change from baseline were observed between female and male subgroups treated with efgartigimod for MG‐ADL (Figure 1A), QMG (Figure 1B), and MG‐QoL15r scores (Figure 1C).

**TABLE 2:** Summary of sex‐specific treatment effects on primary and secondary endpoints in the modified intent‐to‐treat population by sex at birth and treatment.

	Female (*n* = 86)			Male (*n* = 43)			Homogenous between‐sex treatment effect
	Efgartigimod (*n* = 46)	Placebo (*n* = 40)	Treatment effect (efgartigimod vs. placebo)	Efgartigimod (*n* = 19)	Placebo (*n* = 24)	Treatment effect (efgartigimod vs. placebo)	
MG‐ADL responder in Cycle 1 (primary endpoint), *n* (%)	31 (67.4)	13 (32.5)	OR: 4.29 (95% CI, 1.74–10.60)	13 (68.4)	6 (25.0)	OR: 6.50 (95% CI, 1.71–24.77)	*p* = 0.7014
QMG responder in Cycle 1, *n* (%)	26 (56.5)	7 (17.5)	OR: 6.13 (95% CI, 2.25–16.70)	15 (78.9)	2 (8.3)	OR: 41.25 (95% CI, 6.68–254.55)	*p* = 0.1595
Percentage of time having CMI in MG‐ADL score, LS mean (SE)	50.2 (6.6)	26.6 (7.2)	LSMD 23.50 (95% CI, 9.74–37.27)	44.8 (8.9)	26.3 (8.1)	LSMD 18.53 (95% CI, −0.897–37.96)	*p* = 0.6801
Early MG‐ADL responder in Cycle 1, *n* (%)	26 (56.5)	10 (25.0)	OR 3.90 (95% CI, 1.55–9.82)	11 (57.9)	6 (25.0)	OR 4.13 (95% CI, 1.13–15.10)	*p* = 1.0000

Abbreviations: CI, confidence interval; CMI, clinically meaningful improvement; LS, least‐squares; LSMD, least‐squares mean difference; MG‐ADL, Myasthenia Gravis Activities of Daily Living; OR, odds ratio; QMG, Quantitative Myasthenia Gravis; SE, standard error.

**FIGURE 2:**
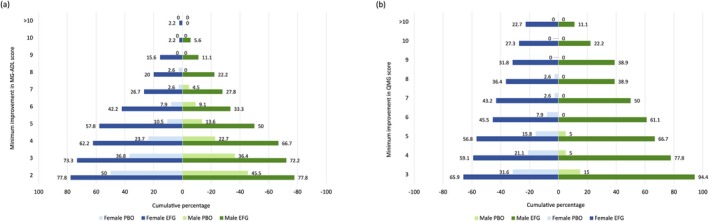
Minimum point improvement in (a) total MG‐ADL score and (b) QMG score during Cycle 1 (Week 4) in the modified intent‐to‐treat population, by sex at birth. EFG, efgartigimod; MG‐ADL, Myasthenia Gravis Activities of Daily Living; PBO, placebo; QMG, Quantitative Myasthenia Gravis.

**FIGURE 3:**
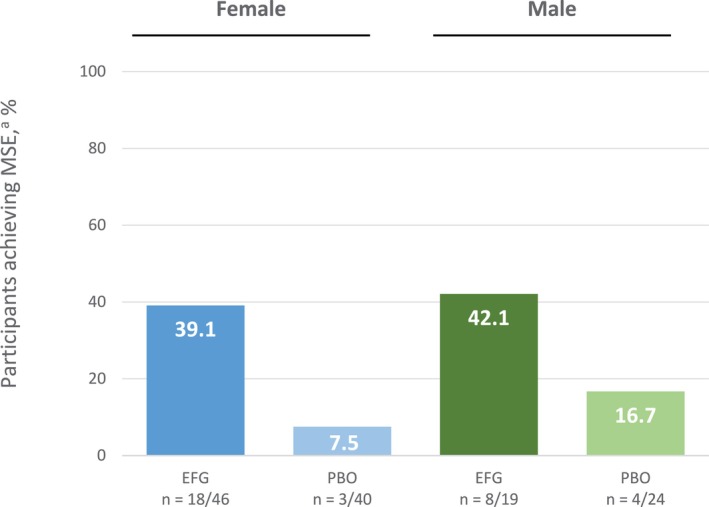
Proportion of participants achieving MSE during the first cycle in the modified intent‐to‐treat population, by sex at birth. ^a^MSE describes achieving an MG‐ADL score of 0 or 1 at any post‐baseline visits within the corresponding cycle. EFG, efgartigimod; MG‐ADL, Myasthenia Gravis Activities of Daily Living; MSE, minimal symptom expression; PBO, placebo.

For both female and male subgroups, a greater proportion of efgartigimod‐treated participants achieved higher levels of improvement in MG‐ADL and QMG scores (up to a > 10‐point reduction) at Week 4 in Cycle 1 compared with participants who received placebo. In the efgartigimod group, 73.3% of female (33/45) and 72.2% (13/18) of male participants achieved a ≥ 3‐point improvement in MG‐ADL score compared with 36.8% (14/38) and 36.4% (8/22) of female and male placebo‐treated participants (Figure 2A). A ≥ 3‐point improvement in QMG score was achieved by 65.9% (29/44) of female and 94.4% (17/18) of male efgartigimod‐treated participants compared with 31.6% (12/38) and 15% (3/20) of female and male placebo‐treated participants, respectively (Figure 2B). There was no statistically significant difference in the proportion of female and male participants achieving MSE during Cycle 1 (*p* = 0.6285) (Figure 3).

### 3.3 Safety

Regardless of sex, efgartigimod was well tolerated, and safety profiles were generally similar between female and male participants (Table 3). Consistent with findings that women in the general population are 30 times more likely to develop a urinary tract infection (UTI) than men [20], a higher rate of UTIs was observed in females compared with males. The safety profile of efgartigimod between female and male participants was consistent with respect to serious TEAEs, serious treatment‐related TEAEs, and Grade ≥ 3 TEAEs, indicating no increased safety concerns for either sex.

**TABLE 3:** Summary of TEAEs in the overall safety population and by sex at birth.

Event, *n* (%)	Female (*n* = 86)		Male (*n* = 43)	
	Efgartigimod (*n* = 46)	Placebo (*n* = 40)	Efgartigimod (*n* = 19)	Placebo (*n* = 24)
Any TEAE	34 (73.9)	33 (82.5)	15 (78.9)	21 (87.5)
Any serious TEAE	2 (4.3)	1 (2.5)	1 (5.3)	5 (20.8)
Any Grade ≥ 3 TEAE	5 (10.9)	2 (5.0)	1 (5.3)	5 (20.8)
Any TEAE of special interest	21 (45.7)	16 (40.0)	8 (42.1)	6 (25.0)
Any infusion‐related reaction	1 (2.2)	3 (7.5)	1 (5.3)	2 (8.3)
Any TEAE deemed treatment related^a^ by the PI	13 (28.3)	10 (25.0)	7 (36.8)	5 (20.8)
Any procedure‐related TEAE	0	0	1 (5.3)	0
Any serious treatment‐related TEAE	1 (2.2)	0	0	0
Any TEAE leading to treatment discontinuation	2 (4.3)	0	0	3 (12.5)
Any TEAE leading to death	0	0	0	0
Most common TEAEs (≥ 10% in any group)				
Headache	13 (28.3)	12 (30.0)	4 (21.1)	5 (20.8)
Upper respiratory tract infection	5 (10.9)	2 (5.0)	4 (21.1)	0
Urinary tract infection	5 (10.9)	2 (5.0)	0	1 (4.2)
Nausea	4 (8.7)	5 (12.5)	1 (5.3)	1 (4.2)
Diarrhea	4 (8.7)	4 (10.0)	1 (5.3)	4 (16.7)
Nasopharyngitis	4 (8.7)	6 (15.0)	5 (26.3)	5 (20.8)
Back pain	1 (2.2)	1 (2.5)	2 (10.5)	0
Myasthenia gravis	1 (2.2)	0	1 (5.3)	3 (12.5)
Hypertension	2 (4.3)	4 (10.0)	1 (5.3)	0

Abbreviations: PI, principal investigator; TEAE, treatment‐emergent adverse event.

^a^ Treatment‐related is defined as at least possibly drug related according to the investigator, or a missing drug relatedness.

### 3.4 Pharmacodynamics

**FIGURE 4:**
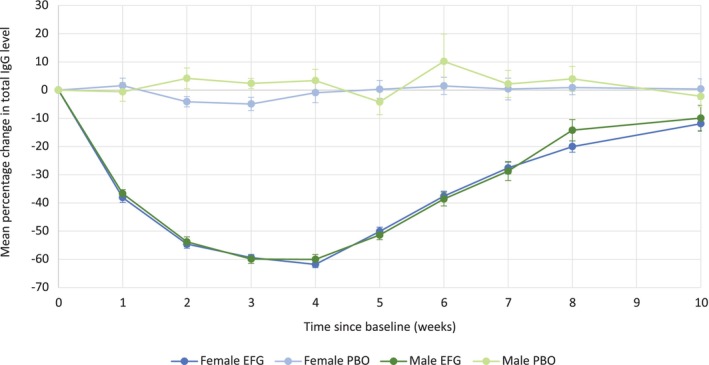
Change in total IgG level during Cycle 1 in the modified intent‐to‐treat population, by sex at birth. Error bars show standard error. EFG, efgartigimod; IgG, immunoglobulin G; PBO, placebo.

No significant difference in efgartigimod pharmacodynamics was observed between female and male study participants, with maximum mean total IgG reductions of 61.8% and 60.0%, respectively, at Week 4 in Cycle 1 (Figure 4).

## 4 Discussion

This post hoc subgroup analysis demonstrated that there were no significant sex‐specific differences in outcomes for AChR‐Ab+ participants with gMG who received efgartigimod or placebo in the Phase 3 ADAPT trial. In line with the primary analysis [12], our findings showed that efgartigimod treatment was well tolerated and resulted in consistent improvements across all primary, secondary, and exploratory gMG‐specific outcome measures, irrespective of sex.

The ADAPT trial consisted of a broad, well‐defined population of participants with gMG. A higher proportion of females had undergone a previous thymectomy compared with male participants (65.1% vs. 44.2%), consistent with previous findings [7]. Age and disease severity are key factors when assessing the clinical decision for thymectomy [20]; the younger age of female participants in the ADAPT study may, in part, account for this observed sex difference in thymectomy rate. However, sensitivity analyses found no significant effect of age and no significant interaction between age and treatment by sex. No significant differences were observed in baseline total MG‐ADL, Myasthenia Gravis Composite (MGC), and MG‐QoL15r scores between female and male participants; however, females did have a significantly higher baseline QMG score than males. Previous studies on sex‐specific differences in disease severity showed mixed results. Whereas Thomsen et al. [7] reported no significant sex differences as measured by baseline MG‐ADL, QMG, MGC, and MG‐QoL15r scores, Wilcke et al. [4] reported that female patients had higher MG‐ADL, QMG, and MG‐QoL15r scores than male patients.

Efgartigimod‐treated participants in both male and female subgroups had a reduction in disease severity compared with placebo, as measured by improvements in MG‐ADL and QMG scores in both Cycle 1 and Cycle 2, which translated into an improved QoL as measured by the improvement in MG‐QoL15r score in Cycle 1. The secondary endpoint of QMG responders in Cycle 1 had a considerably larger odds ratio for treatment effects in comparison to the primary and other secondary endpoints, although the difference in scores was of low magnitude. MMRM for change from baseline in the first 8 weeks during Cycle 1 confirmed there were no sex‐specific treatment effects on this study outcome; the low sample size for male placebo‐treated responders (*n* = 2) and efgartigimod‐treated non‐responders (*n* = 4) was the likely cause of observed differences.

The proportion of male and female participants achieving MSE, an important treatment goal in gMG to ensure adequate disease control and improve participant QoL, was comparable, further highlighting the homogeneous treatment benefit of efgartigimod between female and male subgroups. The consistency in response between sex subgroups observed here is in contrast to that observed by others [7] and may, in some part, result from the weight‐based dosing of efgartigimod. Based on the drug dosing data in ADAPT, there were limited patients/infusions whose efgartigimod dose deviated from 10 mg/kg. Therefore, the average dose in men and women should be closely proportional to the average body weight (10 mg/kg × body weight in kg). The ADAPT study was not designed to assess differences in weight‐based dosing across participant subgroups; however, this may be an area that would benefit from future study.

Efgartigimod was well tolerated, with a between‐sex safety profile comparable to observations in the overall population of the ADAPT study. A similar safety profile was observed between male and female participants. To date, there is limited evidence for sex‐specific differences in placebo and nocebo effects in clinical trials overall [20, 21].

As with many post hoc subgroup analyses, this study was limited by sample size, which was sufficiently powered for the main ADAPT study and not designed to show a difference in the subgroups assessed. Here, limitations are predominantly linked to the small number of participants, especially male participants, included in the subanalysis. This impacted the confidence with which conclusions could be drawn from descriptive analyses in which the number of participants for specific categories was lower and statistical tests for homogeneity of treatment effect between sex subgroups were underpowered. In addition, sex‐specific data were determined based on patient responses to “sex at birth” and the binary choice “male” or “female.” It is possible that some participants chose to report gender identity rather than sex, which could affect study outcomes. This limitation may be addressed in future studies by broadening the patient identification criteria specific to sex and gender. Separately, the large number of different adverse events and the low occurrence for the majority of each adverse event type also meant it was not possible to statistically assess sex‐specific differences for the safety profile of efgartigimod and placebo.

In conclusion, this subanalysis demonstrated that efgartigimod treatment was efficacious and well tolerated, with consistent improvements across gMG‐specific outcome measures irrespective of participant‐reported sex at birth. It provides valuable information to clinicians who must consider differences between the sexes in terms of MG disease course and response to conventional treatment. ADAPT provides clinicians with data showing that efgartigimod‐treated female and male AChR‐Ab+ gMG patients had similar efficacy and safety outcomes.

## Author Contributions

**Sarah Hoffmann:** conceptualization (lead), investigation (equal), methodology (equal), supervision (lead), validation (supporting), visualization, writing – review and editing. **Sihui Zhao:** formal analysis (lead), writing – review and editing (supporting). **Filip Callewaert:** writing – review and editing (supporting). **Silke Schoppe:** writing – review and editing (supporting). **Csilla Rózsa:** writing – review and editing (supporting). **Jennifer Spillane:** writing – review and editing (supporting).

## Funding

The clinical trial and publication were funded by Argenx.

## Consent

All patients provided written informed consent prior to study participation.

## Conflicts of Interest

S.H. has received speaker's honoraria from Alexion Pharmaceuticals, Argenx, Grifols, Johnson & Johnson, Roche and UCB, honoraria for attendance at advisory boards from Alexion Pharmaceuticals, Argenx, Johnson & Johnson, Novartis, and financial research support from Argenx and Johnson & Johnson. S.Z., F.C., and S.S. are full‐time employees of Argenx. C.R. has received speaker's honoraria from AstraZeneca, Medison Pharma and UCB, and served on medical advisory boards for AstraZeneca, Medison Pharma and UCB. J.S. has received speaker's honoraria from Argenx, Johnson & Johnson, and UCB, travel support from Argenx and UCB, and has served on advisory boards for Argenx and UCB.

## Supporting information


**Table S1:** Summary of sex‐specific treatment effects in the AChR‐Ab+ modified intent‐to‐treat population during the second treatment cycle by sex at birth and treatment.
**Table S2:** MMRM for QMG change from baseline of the first 8 weeks during Cycle 1 in the AChR‐Ab+ mITT population by sex at birth (between‐sex comparison).
**Table S3:** MMRM for QMG change from baseline of the first 8 weeks during Cycle 1 in the AChR‐Ab+ mITT population by sex at birth (between‐sex comparison).

## Data Availability

Data are available on request due to privacy/ethical restrictions. The datasets presented in this article are not readily available but can be requested by qualified researchers who engage in rigorous independent scientific research and can be provided after review and approval of a research proposal and statistical analysis plan and execution of a data sharing agreement. Data requests can be submitted at any time and the data will be accessible for 12 months. Argenx is committed to responsible data sharing regarding the clinical trials they fund. Included in this commitment is access to anonymized individual and trial‐level data (analysis datasets), and other information (e.g., protocols and clinical study reports), as long as the trials are not part of an ongoing or planned regulatory submission. This includes requests for clinical trial data for unlicensed products and indications. Requests to access the datasets should be directed to esr@argenx.com.
