# Supplementary material for: Post Hoc, Sex‐Specific Subgroup Analysis of Efgartigimod in Patients With Generalized Myasthenia Gravis From the ADAPT Trial: A Sex and Gender Equity in Research (SAGER) Guidelines Approach
Source: Muscle Nerve. 2026 Jan 10;73(4):566–74. doi: 10.1002/mus.70135 (PMC12969971; doi:10.1002/mus.70135)
Supplement: Supplementary file 1 — Table S1: Summary of sex‐specific treatment effects in the AChR‐Ab+ modified intent‐to‐treat population during the second treatment cycle by sex at birth and treatment. Table S2: MMRM for QMG change from baseline of the first 8 weeks during Cycle 1 in the AChR‐Ab+ mITT population by sex at birth (between‐sex comparison). Table S3: MMRM for QMG change from baseline of the first 8 weeks during Cycle 1 in the AChR‐Ab+ mITT population by sex at birth (between‐sex comparison). [file MUS-73-566-s001.docx]

**Supplemental Information for**

**Post-hoc, sex-specific subgroup analysis of efgartigimod in patients with generalised myasthenia gravis from the ADAPT trial: A Sex and Gender Equity in Research (SAGER) guidelines approach**

Prof. Dr. Sarah Hoffmann^1^, Sihui Zhao^2^, Filip Callewaert, PhD^2^, Silke Schoppe^2^, Csilla Rózsa, MD PhD^3*^, Jennifer Spillane, MCRCP PhD^4*^

^1^Charité-Universitätsmedizin, corporate member of Freie Universität Berlin and Humboldt-Universität zu Berlin, Department of Neurology, Neuroscience Clinical Research Center (NCRC) and Integrated Myasthenia Gravis Center, Charitéplatz 1, 10117 Berlin, Germany

^2^argenx, Ghent, Belgium

^3^Department of Neurology, Jahn Ferenc Dél-pesti Hospital, Budapest, Hungary

^4^Centre for Neuromuscular Disease, National Hospital for Neurology and Neurosurgery Queen Square, University College London Hospitals (UCLH) National Health Service (NHS) Foundation Trust, London, United Kingdom

*Both authors contributed equally.

# TABLE S1 Summary of sex-specific treatment effects in the AChR-Ab+ modified intent-to-treat population during the second treatment cycle by sex at birth and treatment

|  | **Female (*n* = 60)** | |  | **Male (*n* = 34)** | |  |  |
| --- | --- | --- | --- | --- | --- | --- | --- |
|  | **Efgartigimod (*n* = 32)** | **Placebo  (*n* = 28)** | **Treatment effect**  **(efgartigimod vs placebo)** | **Efgartigimod (*n* = 19)** | **Placebo  (*n* = 15)** | **Treatment effect**  **(efgartigimod vs placebo)** | **Homogenous between-sex treatment effect** |
| MG-ADL responder, *n* (%) | 21 (65.6) | 9 (32.1) | OR: 4.03 (95% CI, 1.37–11.84) | 15 (78.9) | 2 (13.3) | OR: 24.38 (95% CI, 3.82–155.45) | *p* = .1583 |
| QMG responder,  *n* (%) | 15 (46.9) | 5 (17.9) | OR: 4.06 (95% CI, 1.23–13.35) | 9 (47.4) | 0 (0.0) | OR: 28.05 (95% CI, 1.47–535.74) | *p* = .2398 |
| Early MG-ADL responder, *n* (%) | 20 (62.5) | 6 (21.4) | OR 6.11 (95% CI, 1.93–19.34) | 14 (73.7) | 2 (13.3) | OR 18.20 (95% CI, 2.99–110.68) | *p* = 1.0000 |

Abbreviations: AChR-Ab+, acetylcholine receptor antibody–positive; CI, confidence interval; MG-ADL, Myasthenia Gravis Activities of Daily Living; OR, odds ratio; QMG, Quantitative Myasthenia Gravis; SE, standard error.

# TABLE S2 MMRM for QMG change from baseline of the first 8 weeks during cycle 1 in the AChR-Ab+ mITT population by sex at birth (between-sex comparison)

|  |  |  | **LSM** | | | **LSMD** | | |
| --- | --- | --- | --- | --- | --- | --- | --- | --- |
| **Visit** | **Treatment** | **Sex** | **Estimate** | **SE** | **95% CI** | **Estimate** | **SE** | ***P*-value** |
| Week 1 | EFG | F | -2.23 | 0.61 | (-3.44; -1.02) | 0.56 | 0.87 | 0.5253 |
|  |  | M | -2.78 | 0.86 | (-4.48; -1.09) |  |  |  |
|  | PBO | F | 0.41 | 0.66 | (-0.91; 1.72) | -0.15 | 0.87 | 0.8649 |
|  |  | M | 0.55 | 0.82 | (-1.07; 2.18) |  |  |  |
| Week 2 | EFG | F | -4.24 | 0.66 | (-5.54; -2.94) | 0.02 | 0.99 | 0.9829 |
|  |  | M | -4.26 | 0.94 | (-6.12; -2.40) |  |  |  |
|  | PBO | F | -0.38 | 0.72 | (-1.79; 1.04) | -0.91 | 0.98 | 0.3527 |
|  |  | M | 0.54 | 0.90 | (-1.23; 2.30) |  |  |  |
| Week 3 | EFG | F | -4.73 | 0.69 | (-6.10; -3.36) | 0.84 | 1.06 | 0.4282) |
|  |  | M | -5.57 | 1.00 | (-7.54; -3.60) |  |  |  |
|  | PBO | F | -0.30 | 0.75 | (-1.78; 1.19) | -0.16 | 1.06 | 0.8786 |
|  |  | M | -0.14 | 0.96 | (-2.03; 1.76) |  |  |  |
| Week 4 | EFG | F | -5.50 | 0.71 | (-6.90; -4.10) | 0.93 | 1.10 | 0.3980 |
|  |  | M | -6.43 | 1.03 | (-8.46; -4.40) |  |  |  |
|  | PBO | F | -0.64 | 0.77 | (-2.16; 0.88) | -0.30 | 1.09 | 0.7811 |
|  |  | M | -0.34 | 0.98 | (-2.27; 1.59) |  |  |  |
| Week 5 | EFG | F | -5.35 | 0.75 | (-6.82; -3.87) | 0.63 | 1.18 | 0.5928 |
|  |  | M | -5.98 | 1.08 | (-8.12; -3.83) |  |  |  |
|  | PBO | F | -0.76 | 0.81 | (-2.36; 0.83) | -0.68 | 1.17 | 0.5658 |
|  |  | M | -0.09 | 1.04 | (-2.15; 1.97) |  |  |  |
| Week 6 | EFG | F | -4.59 | 0.70 | (-5.96; -3.21) | -0.49 | 1.08 | 0.6476 |
|  |  | M | -4.09 | 1.01 | (-6.08; -2.10) |  |  |  |
|  | PBO | F | -0.63 | 0.76 | (-2.14; 0.87) | -0.39 | 1.07 | 0.7180 |
|  |  | M | -0.25 | 0.97 | (-2.16; 1.66) |  |  |  |
| Week 7 | EFG | F | -3.85 | 0.69 | (-5.21; -2.50) | -0.11 | 1.05 | 0.9167 |
|  |  | M | -3.74 | 0.99 | (-5.70; -1.79) |  |  |  |
|  | PBO | F | -0.93 | 0.75 | (-2.41; 0.54) | -0.86 | 1.04 | 0.4147 |
|  |  | M | -0.08 | 0.95 | (-1.94; 1.79) |  |  |  |
| Week 8 | EFG | F | -2.65 | 0.62 | (-3.87; -1.43) | -0.73 | 0.90 | 0.4187 |
|  |  | M | -1.92 | 0.88 | (-3.66; -0.18) |  |  |  |
|  | PBO | F | -0.84 | 0.68 | (-2.18; 0.50) | -0.35 | 0.91 | 0.7018 |
|  |  | M | -0.49 | 0.86 | (-2.19; 1.21) |  |  |  |

AChR-Ab+, acetylcholine receptor antibody-positive; EFG, efgartigimod; LSM, least-squares mean; LSMD, least-squares mean difference; mITT, modified intent-to-treat; MMRM, mixed model with repeated measures; PBO, placebo; SE, standard error.

# TABLE S3 MMRM for QMG change from baseline of the first 8 weeks during cycle 1 in the AChR-Ab+ mITT population by sex at birth (between-sex comparison)

|  |  |  | **LSM** | | | **LSMD** | | |
| --- | --- | --- | --- | --- | --- | --- | --- | --- |
| **Visit** | **Sex** | **Treatment** | **Estimate** | **SE** | **95% CI** | **Estimate** | **SE** | ***P*-value** |
| Week 1 | F | EFG | -2.23 | 0.61 | (-3.44; -1.02) | -2.63 | 0.69 | 0.0002 |
|  |  | PBO | 0.41 | 0.66 | (-0.91; 1.72) |  |  |  |
|  | M | EFG | -2.78 | 0.86 | (-4.48; -1.09) | -3.34 | 1.01 | 0.0013 |
|  |  | PBO | 0.55 | 0.82 | (-1.07; 2.18) |  |  |  |
| Week 2 | F | EFG | -4.24 | 0.66 | (-5.54; -2.94) | -3.86 | 0.78 | <0.0001 |
|  |  | PBO | -0.38 | 0.72 | (-1.79; 1.04) |  |  |  |
|  | M | EFG | -4.26 | 0.94 | (-6.12; -2.40) | -4.79 | 1.14 | <0.0001 |
|  |  | PBO | 0.54 | 0.90 | (-1.23; 2.30) |  |  |  |
| Week 3 | F | EFG | -4.73 | 0.69 | (-6.10; -3.36) | -4.43 | 0.84 | <0.0001 |
|  |  | PBO | -0.30 | 0.75 | (-1.78; 1.19) |  |  |  |
|  | M | EFG | -5.57 | 1.00 | (-7.54; -3.60) | -5.44 | 1.23 | <0.0001 |
|  |  | PBO | -0.14 | 0.96 | (-2.03; 1.76) |  |  |  |
| Week 4 | F | EFG | -5.50 | 0.71 | (-6.90; -4.10) | -4.85 | 0.87 | <0.0001 |
|  |  | PBO | -0.64 | 0.77 | (-2.16; 0.88) |  |  |  |
|  | M | EFG | -6.43 | 1.03 | (-8.46; -4.40) | -6.09 | 1.27 | <0.0001 |
|  |  | PBO | -0.34 | 0.98 | (-2.27; 1.59) |  |  |  |
| Week 5 | F | EFG | -5.35 | 0.75 | (-6.82; -3.87) | -4.58 | 0.94 | <0.0001 |
|  |  | PBO | -0.76 | 0.81 | (-2.36; -0.83) |  |  |  |
|  | M | EFG | -5.98 | 1.08 | (-8.12; -3.83) | -5.89 | 1.37 | <0.0001 |
|  |  | PBO | -0.09 | 1.04 | (-2.15; 1.97) |  |  |  |
| Week 6 | F | EFG | -4.59 | 0.70 | (-5.96; -3.21) | -3.95 | 0.85 | <0.0001 |
|  |  | PBO | -0.63 | 0.76 | (-2.14; 0.87) |  |  |  |
|  | M | EFG | -4.09 | 1.01 | (-6.08; -2.10) | -3.85 | 1.25 | 0.0026 |
|  |  | PBO | -0.25 | 0.97 | (-2.16; 1.66) |  |  |  |
| Week 7 | F | EFG | -3.85 | 0.69 | (-5.21; -2.50) | -2.92 | 0.83 | 0.0006 |
|  |  | PBO | -0.93 | 0.75 | (-2.41; 0.54) |  |  |  |
|  | M | EFG | -3.74 | 0.99 | (-5.70; -1.79) | -3.67 | 1.22 | 0.0032 |
|  |  | PBO | -0.08 | 0.95 | (-1.94; 1.79) |  |  |  |
| Week 8 | F | EFG | -2.65 | 0.62 | (-3.87; -1.43) | -1.81 | 0.71 | 0.0123 |
|  |  | PBO | -0.84 | 0.68 | (-2.18; 0.50) |  |  |  |
|  | M | EFG | -1.92 | 0.88 | (-3.66; -0.18) | -1.43 | 1.05 | 0.1762 |
|  |  | PBO | -0.49 | 0.86 | (-2.19; 1.21) |  |  |  |

AChR-Ab+, acetylcholine receptor antibody-positive; EFG, efgartigimod; LSM, least-squares mean; LSMD, least-squares mean difference; mITT, modified intent-to-treat; MMRM, mixed model with repeated measures; PBO, placebo; SE, standard error.
